# ﻿Two new species of *Craterellus* (Cantharellales, Hydnaceae) with veined hymenophore from north-eastern China

**DOI:** 10.3897/mycokeys.91.84730

**Published:** 2022-07-19

**Authors:** Gui-Ping Zhao, Jia-Jun Hu, Yong-Lan Tuo, Yu Li, Bo Zhang

**Affiliations:** 1 Engineering Research Center of Edible and Medicinal Fungi, Ministry of Education, Jilin Agricultural University, Changchun, Jilin 130118, China Jilin Agricultural University Changchun China

**Keywords:** Chinese species, molecular phylogeny, morphology

## Abstract

In this contribution to the genus *Craterellus* in northern China, two new species are introduced: *Craterellusconnatus* and *C.striatus*. These species and *C.atrobrunneolus*, initially described in south-western China, are highly similar and closely related. The species delimitation is molecularly supported by multigene phylogenetic analysis of the nr LSU and *tef*-1α region. *Craterellusconnatus* is characterised by its medium-sized basidiomata, greyish-brown and smooth pileus with an off-white margin, the hymenophore with a strongly anastomosing vein, turning khaki upon drying, connate stipe, broad ellipsoid to ellipsoid basidiospores (6.1–7.8 × 4.8–5.9 μm), slender basidia with (2)4–6 sterigmata and the absence of clamp connection. *Craterellusstriatus* is characterised by its small-sized basidiomata, fibrillose, greyish-brown to yellowish-brown, fully perforated pileus with a brown fringe, the hymenophore with a forking vein, the stipe inflated at the base, broad ellipsoid to ellipsoid basidiospores (6.8–8.0 × 5.1–6.0 μm), 2–6 spored basidia, encrusted hyphae and the absence of clamp connection. Detailed macroscopic and microscopic descriptions, accompanied by illustrations and a taxonomic discussion, are presented. A key to the Chinese *Craterellus* species is also provided.

## ﻿Introduction

*Craterellus* Pers., typified with *C.cornucopioides* (L.) Pers. (Persoon 1825), belongs to *Hydnaceae* of *Cantharellales* (Hibbett et al. 2014). It is well represented in northern temperate zones and occurs in the tropics (Dahlman et al. 2000). Species in this genus are characterised by funnel-shaped fruiting bodies, hollow stipes, usually dark grey to black or yellow pileus (Corner 1966). *Craterellus* is an important genus of wild edible mushrooms and is renowned for its high economic, medicinal and ecological values (Pilz 2003). Approximately 162 records of *Craterellus* (including infraspecific taxa) have been found in Index Fungorum (http://www.indexfungorum.org). However, most of these species have been transferred to other genera, based on a phylogenetic analysis of the large nuclear subunit (nr LSU) and the internal transcribed spacer region (ITS), including *Cantharellus* Adans. ex Fr., *Gomphus* Pers. and *Polyozellus* Murrill etc. (Dahlman et al. 2000; Contu et al. 2009; Olariaga et al. 2009; Wilson et al. 2012; Montoya et al. 2021). Nearly 100 names have been considered legitimate in MycoBank (https://www.mycobank.org/) to date.

In China, species diversity, taxonomy and phylogeny of macrofungi have been investigated in recent years, many new species having been discovered (Zhong et al. 2018; Cui et al. 2019; Sun et al. 2020; Zhang et al. 2020a; Cao et al. 2021a, b; Liu et al. 2021, 2022a, b; Ji et al. 2022; Sun et al. 2022). *Craterellus* is one of the important fungal genera of macrofungi, 14 species of *Craterellus* having been recorded in China: *C.albidus* Chun Y. Deng, M. Zhang & J. Zhang, *C.atratus* (Corner) Yomyart, Watling, Phosri, Piap. & Sihan., *C.atrobrunneolus* T. Cao & H.S. Yuan, *C.aureus* Berk. & M.A. Curtis, *C.badiogriseus* T. Cao & H.S. Yuan, *C.cornucopioides*, *C.croceialbus* T. Cao & H.S. Yuan, *C.lutescens* (Fr.) Fr., *C.luteus* T.H. Li & X.R. Zhong, *C.macrosporus* T. Cao & H.S. Yuan, *C.odoratus* (Schwein.) Fr., *C.sinuosus* (Fr.) Fr., *C.squamatus* T. Cao & H.S. Yuan, *C.tubaeformis* (Fr.) Quél (Bi 1994; Mao 1998; Dai et al. 2010; Gu et al. 2012; Li et al. 2015; Zhong et al. 2018; Zhang et al. 2020a; Cao et al. 2021a, b). Nevertheless, due to their less attractive appearance, which can be confused with the environment, it is challenging to find *Craterellus* species in the field, causing them to be overlooked and thus remain undescribed. In addition, some species of *Craterellus*, especially those with the non-perforated pileus, are easily confused with other morphologically similar taxa, for example,, some species of *Clitocybe* (Fr.) Staude, *Gomphus* or *Polyozellus*, making it never easily distinguished from others in fieldwork.

During a recent survey of macrofungi in northern China, we discovered some exciting and novel species of *Craterellus*. In this contribution, the subsequent morphological and molecular analyses of the transcription elongation factor 1-alpha (*tef*-1α) and nr LSU sequences represented two new species, *C.connatus* G. P. Zhao, J. J. Hu, B. Zhang & Y. Li and *C.striatus* G. P. Zhao, J. J. Hu, B. Zhang & Y. Li which are described and illustrated herein. A key of *Craterellus* in China is provided as well.

## ﻿Materials and methods

### ﻿Vouchers and morphological analyses

This paper is principally based on materials collected by the senior author and collaborators over the past four years in northern China and specimens have been deposited at the Mycological Herbarium of Jilin Agricultural University (**HMJAU**).

Photographs and descriptions of macroscopic morphological characteristics were made from fresh materials in the field. The colours correspond to the “Flora of British fungi: colour identification chart” (Royal Botanic Garden 1969). Collections were dried in an oven at 45 °C and rehydrated with 95% alcohol. All microscopic observations and measurements were made in ammoniacal Congo red with a 5% aqueous potassium hydroxide (KOH) solution to improve tissue dissociation and matrix dissolution. Melzer’s Reagent was also used in the study. Measurements of basidiospores cite length and length/width ratio (Q) in this format: (minimum–) mean minus standard deviation–mean value–mean plus standard deviation (–maximum measured), Q_m_ = average Q of all basidiospores measured ± sample standard deviation; spore measurements are based on 20 spores. Microscopic features were examined with the aid of the ZEISS Axio Lab A1.

### ﻿DNA extraction, amplification and sequencing

Genomic DNA was extracted from dried or fresh material stored in desiccant silica gel. The extraction method followed the nuclear Plant Genomic DNA Kit (Kangwei Century Biotechnology Company Limited, Beijing, China). The amplified segments were the nr LSU and the *tef*-1α regions. The nr LSU region was amplified using LR0R and LR5 (Vilgalys and Hester 1990). The *tef*-1α region was amplified using tefF and tefR (Morehouse et al. 2003). Polymerase chain reaction (PCR) amplifications were performed in a total volume of 25 μl containing 1 μl template DNA, 1 μl of each primer, 10.5 μl distilled water and 12.5 μl PCR mix (2× Es Taq MasterMix, CWBIO, China). PCR amplification conditions followed Vilgalys and Hester (1990) for the nr LSU region and Morehouse et al. (2003) for the *tef*-1α region. The PCR products were subjected to electrophoresis on 1% agarose gel. Sequencing was performed by Sangon Biotech (Shanghai, China) using the same primer pairs used for the PCR.

### ﻿Phylogenetic analyses

This study is based on around 25 specimens, including two outgroups (*Hydnumellipsosporum* Ostrow & Beenken and *Cantharelluscibarius* Fr.) (Bijeesh et al. 2018; Zhong et al. 2018; Zhang et al. 2020a; Cao et al. 2021b). Sequences of the nr LSU and *tef*-1α regions were newly produced in this study and downloaded from GenBank (http://www.ncbi.nlm.nih.gov/). Detailed sample information is provided in Table 1.

**Table 1.** Information on the specimens that were used in the phylogenetic analyses. Sequences that were newly generated in this study are indicated in black bold. T: Type.

Taxon	Voucher number	Country	GenBank accession number		References
			nr LSU	*tef*-1α	
*Craterellusalbidus*	HGASMF013581 (T)	China	MT921161	–	Zhang et al. (2020a)
*C.albidus*	HGASMF0110046	China	MT921162		Zhang et al. (2020a)
*C.albostrigosus*	CAL1624 (T)	India	MG593194	–	Bijeesh et al. (2018)
*C.atratoides*	TH9232 (T)	Guyana	NG042660	–	Wilson et al. (2012)
*C.atratoides*	MCA1313	Guyana	JQ915119	–	Wilson et al. (2012)
*C.atratus*	MCA1070	Guyana	JQ915118	–	Wilson et al. (2012)
*C.atratus*	TH9203	Guyana	JQ915133	–	Wilson et al. (2012)
*C.atrobrunneolus*	Yuan13878 (T)	China	MN894058	–	Cao et al. (2021b)
*C.badiogriseus*	Yuan 14776 (T)	China	MW979532	MW999432	Cao et al. (2021a)
*C.badiogriseus*	Yuan 14779	China	MW979533	MW999433	Cao et al. (2021a)
*C.caeruleofuscus*	MH17001	USA	MT237468	–	Genbank
*C.cinereofimbriatus*	TH9075 (T)	Guyana	JQ915131	–	Wilson et al. (2012)
*C.cinereofimbriatus*	TH8999	Guyana	JQ915130	–	Wilson et al. (2012)
*C.cinereofimbriatus*	TH9264	Guyana	JQ915138	–	Wilson et al. (2012)
***C.connatus***	**HMJAU 61462 (T)**	**China**	**OM509448**	**ON125915**	**This study**
***C.connatus***	**HMJAU 61462**	**China**	–	**ON125916**	**This study**
*C.cornucopioides*	HbO53302	Norway	AF105301	–	Dahlman et al. (2000)
*C.cornucopioides*	UPSF11801	USA	AF105299	–	Dahlman et al. (2000)
*C.excelsus*	MCA3107	Guyana	JQ915121	–	Wilson et al. (2012)
*C.excelsus*	TH7515	Guyana	JQ915127	–	Wilson et al. (2012)
*C.fallax*	AFTOL286	USA	AY700188	–	Genbank
*C.ignicolor*	UPSF11794	USA	AF105314	–	Dahlman et al. (2000)
*C.indicus*	PUN3884 (T)	India	HM113529	–	Kumari et al. (2012)
*C.indicus*	MSR7	India	HQ450770		Kumari et al. (2012)
*C.indicus*	MSR8	India	HQ450771		Kumari et al. (2012)
*C.inusitatus*	CAL 1625 (T)	India	MG593195	–	Bijeesh et al. (2018)
*C.lutescens*	UPSF11789	Sweden	AF105302	–	Dahlman et al. (2000)
*C.lutescens*	UPSF11790	Sweden	AF105303	–	Dahlman et al. (2000)
*C.lutescens*	UPSF11791	Spain	AF105304	–	Dahlman et al. (2000)
*C.luteus*	GDGM46432	China	MG727898	–	Zhong et al. (2018)
*C.luteus*	GDGM48105 (T)	China	MG701171	–	Zhong et al. (2018)
*C.luteus*	GDGM49495	China	MG806926	–	Zhong et al. (2018)
***C.striatus***	**HMJAU 61463 (T)**	**China**	**OM509446**	**ON125913**	**This study**
***C.striatus***	**HMJAU 61463**	**China**	**OM509447**	**ON125914**	**This study**
*C.odoratus*	UPSF11794	USA	AF105306	–	Dahlman et al. (2000)
*C.olivaceoluteus*	TH9205 (T)	Guyana	JQ915135	–	Wilson et al. (2012)
*C.olivaceoluteus*	MCA3186	Guyana	JQ915124	–	Wilson et al. (2012)
*C.parvogriseus*	CAL1533 (T)	India	MF421098	–	Das et al. (2017)
*C.parvogriseus*	CAL1534	India	NG059049	–	Das et al. (2017)
*C.pleurotoides*	TH9220 (T)	Guyana	JQ915136	–	Wilson et al. (2012)
*C.pleurotoides*	MCA3124	Guyana	JQ915123	–	Wilson et al. (2012)
*C.strigosus*	MAC1750	Guyana	JQ915120	–	Wilson et al. (2012)
*C.strigosus*	TH9204 (T)	Guyana	JQ915134	–	Wilson et al. (2012)
*C.tubaeformis*	UPSF11793	Sweden	AF105307	–	Dahlman et al. (2000)
*C.tubaeformis*	BB 07.293	Slovakia	KF294640	GQ914989	Buyck and Hofstetter (2011)
*Cantharelluscibarius*	BIO 10986 (T)	Sweden	KR677539	KX828823	Olariaga et al. (2017)
*Hydnumellipsosoporxum*	FD3281	Switzerland	KX086217	–	Genbank

Sequences were assembled and edited using the software package Sequencher 5.4.6 (Gene Codes Corp., USA). Alignment of sequence data was performed with MUSCLE in MEGA 7.0.21 (Kumar et al. 2016). For Bayesian Inference (BI) analyses, the most suitable substitution model for each gene partition was calculated with ModelFinder (Kalyaanamoorthy et al. 2017) in PhyloSuite 1.2.2. (Zhang et al. 2020b). BI analyses were performed using MrBayes in PhyloSuite 1.2.2 (Zhang et al. 2020b), implementing the Markov Chain Monte Carlo (MCMC) technique. Four simultaneous Markov chains were run from random trees, keeping one tree every 200^th^ generation until the average standard deviation of split frequencies was below 0.01. The Maximum Likelihood analysis was performed using RAxML-HPC2 on XSEDE 8.2.12 (Stamatakis 2014) in the CIPRES Science Gateway portal (https://www.phylo.org/portal2/tools.action) with the GTRGAMMA model and searching for the most likely tree with 1000 heuristic replicates. The bootstrap support (BS) of ≥ 50% in the ML tree and BPP of ≥ 0.75 indicated statistical significance. The phylogenetic trees were visualised using FigTree 1.4.23 (Andrew 2016).

## ﻿Results

### ﻿Phylogeny

**Figure 1.**
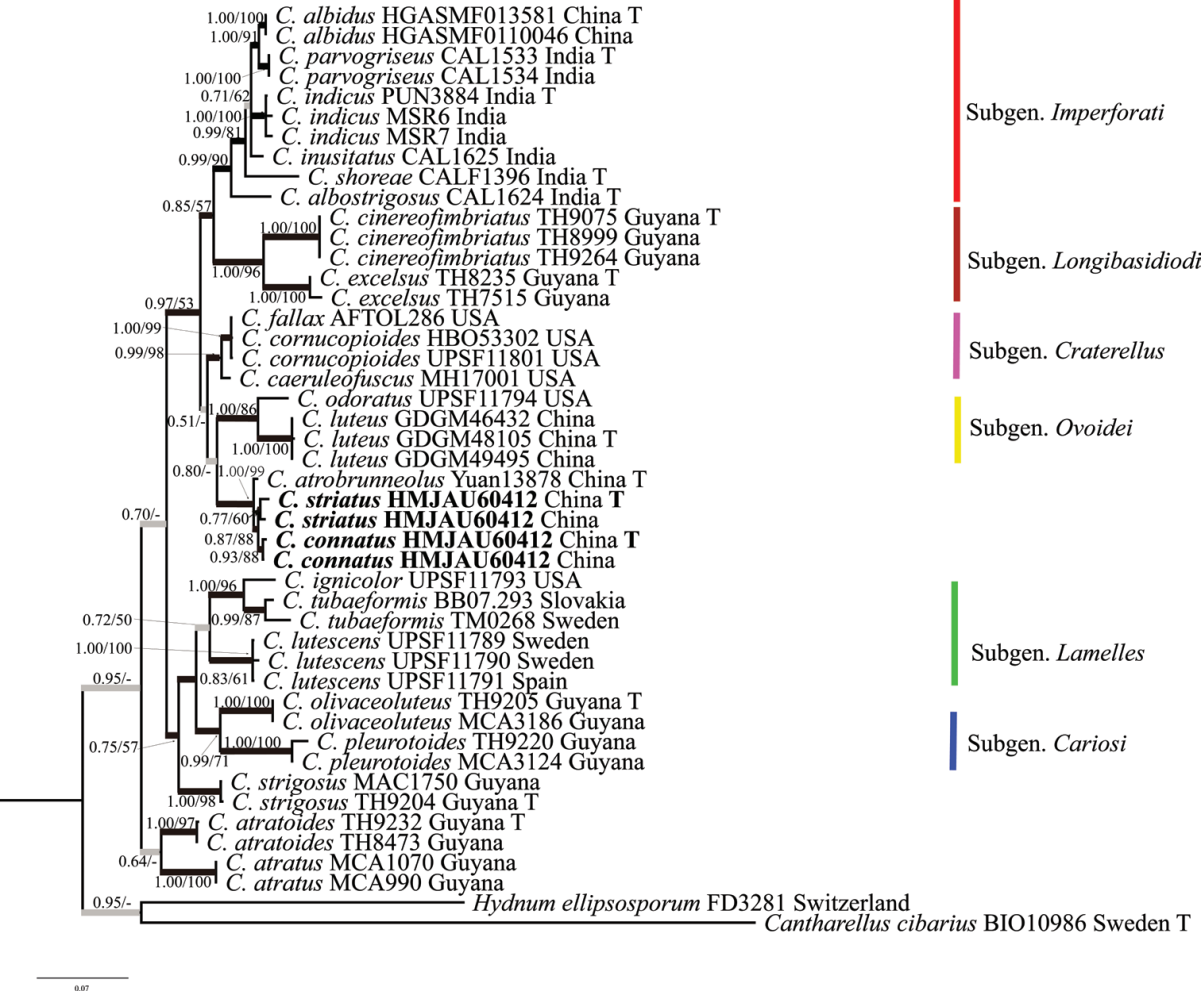
BI best tree inferred from the nr LSU and *tef*-1α region analysis for 25 specimens. Branches that received both bootstrap support (MLBS) ≥ 50% and Bayesian posterior probabilities (PP) ≥ 0.75 are in bold; branches supported by either MLBS or BPP are in grey. Both values (MLBS/BPP) are reported along the branches. Taxon names shown in bold indicate the specimens examined in this study.

Seven new DNA sequences (3 nr LSU, 4 *tef*-1α) were produced for this study. After removing introns and low-homology regions, the final combined alignment of these two genes totalled 2,027 characters (nr LSU: 1,024 characters; *tef*-1α: 1,003 characters). The best models for the BI analysis of the concatenated dataset were SYM+I+G4 for nr LSU and SYM+G4 for tef-1α. The most likely tree inferred by ML analysis of the combined dataset exhibited a quite similarly supported topology as the Bayesian majority-rule consensus tree with an average standard deviation of split frequencies = 0.005143. The most likely tree, based on 1000 searches, is depicted in Fig. 1 with associated bootstrap values. Maximum Likelihood Bootstrap (MLBS) and Bayesian Posterior Probability (BPP) were established along the branches. Phylogenetic analyses show (Fig. 1) that our two new species, *C.connatus* and *C.striatus* were clustered together with *C.atrobrunneolus* in a monophyletic clade (MLBS = 99%, BPP = 1.00). *C.connatus* (MLBS = 88%, BPP = 0.93) formed sister relationships (MLBS = 88%, BPP = 0.87) with *C.striatus* (MLBS = 60%, BPP = 0.77).

### ﻿Taxonomy

#### ﻿ Craterellus connatus

Taxon classificationFungiCantharellalesCantharellaceae

G.P. Zhao, J.J. Hu, B. Zhang & Y. Li sp. nov.

A10BB824-F0E9-53BF-9F4E-B6E9189F50EC

 842527

Figs 2 , 3

##### Holotype.

China. Jilin Province, Jilin City, Jiaohe County, Lafashan National Forest Part, Red Leaves Canyon, alt. 802.5 m, 43.75°N, 127.10°E, 5 September 2018, Bo Zhang HMJAU 61462, GenBank Acc. nos.: nr LSU = OM509448, *tef*-1α = ON125915, ON125916).

**Figure 2.**
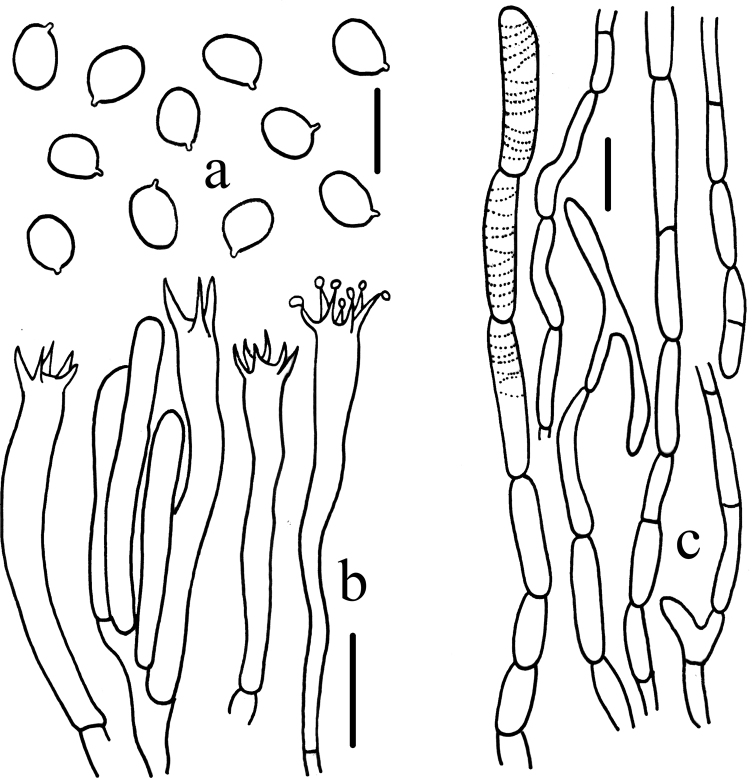
*Craterellusconnatus* (HMJAU 60411, holotype) **A** fresh basidiocarps **B** connate stipes **C** margin of pileus **D** hymenophore. Scale bars: 1 cm (**A, B**)

##### Etymology.

*Connatus*: referring to several stipes grown together from the base upwards.

##### Diagnosis.

Differs from other *Craterellus* species by its greyish-brown and smooth pileus with an off-white margin, hymenophore with a strongly anastomosing vein and the colouration turning khaki upon drying.

**Figure 3.**
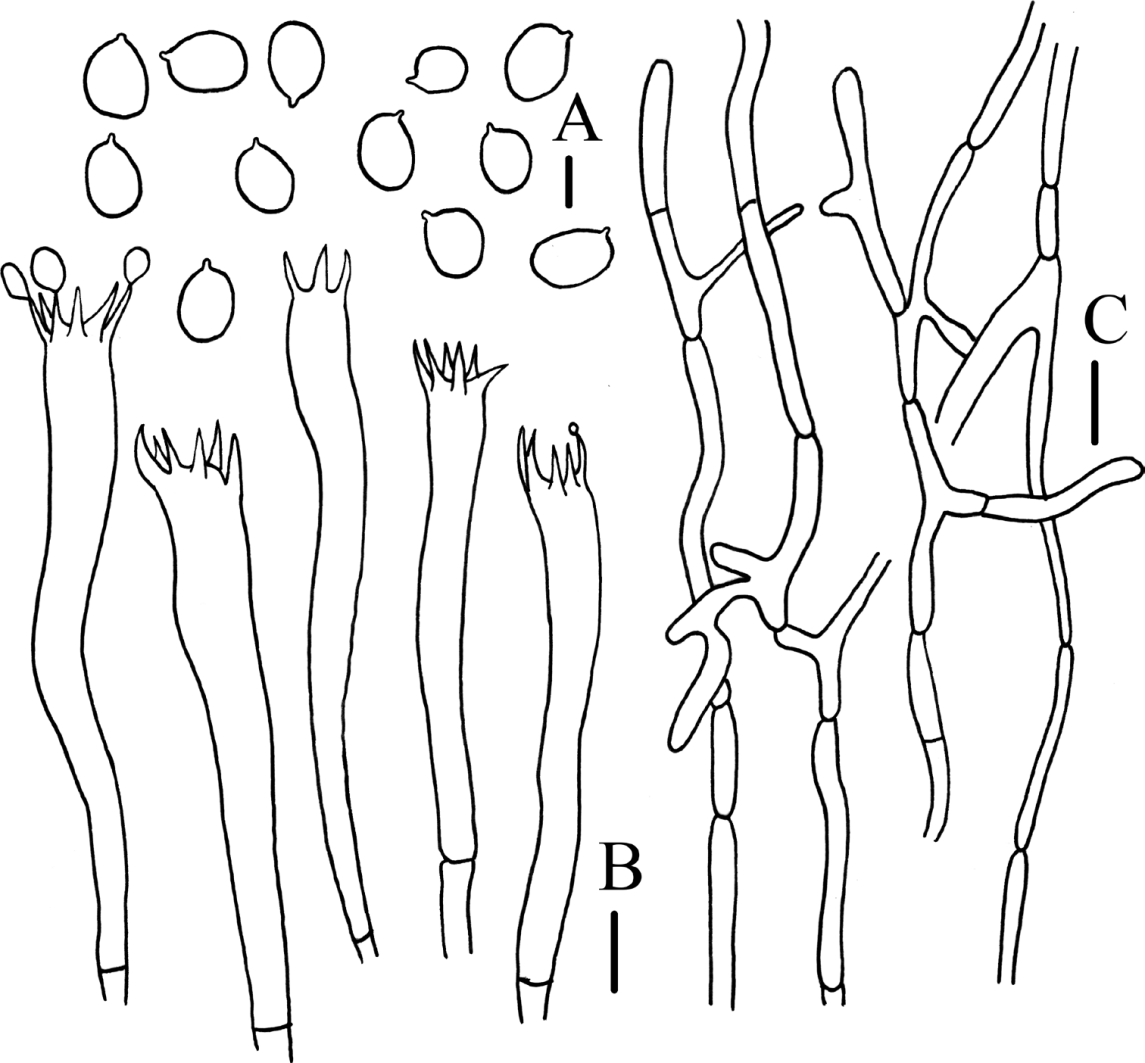
Microscopic characteristics of *Craterellusconnatus* (HMJAU 60411) **A** basidiospores **B** basidia **C** pileipellis. Scale bars: 5 μm (**A**); 10 μm (**B**); 20 μm (**C**).

##### Description.

***Pileus*** 18–30 mm in diam., infundibuliform, deeply depressed at the centre, perforated continuously to the base of the stipe; margin inrolled when young, then expand upwards, becoming upturned finally, broadly wavy; surface greyish-brown in the centre, the marginal edge dirty white, smooth. ***Hymenophore*** consists of longitudinal, anastomosing veined, off-white, turning khaki when drying and decurrent. ***Stipe*** 25–30 × 3 mm, equal to subcylindrical, hollow, central, smooth, greyish-brown. ***Text*** fleshy, greyish-brown. ***Odour*** light. ***Taste*** unknown. ***Spore print*** not obtained.

***Basidiospores*** 6.1–6.9–7.8(8.1) × 4.8–5.3–5.9 μm, Q = (1.16)1.20–1.32–1.45(1.56), Q_m_ = 1.32 ± 0.09, broad ellipsoid to ellipsoid, smooth, thin-walled, pale yellow in 5% aqueous KOH, inamyloid. ***Basidia*** (40)50–72.5(75) × (6)7.5–8(10) μm, clavate, sterigmata (2)4-6. ***Cystidia*** absent. ***Hymenium*** in transverse section 125–175 μm thick, yellowish-brown in 5% aqueous KOH. ***Pileipellis*** scarcely differentiated from the trama, a cutis of vastly inflated hyphae, yellowish-brown in 5% aqueous KOH; individual hyphae (22)27–63(64) × 6–15 μm, thin-walled, branched occasionally, secondary septation absent, hyaline in 5% aqueous KOH. ***Pileus trama*** up to 500 μm thick, subparallel, yellowish-brown in 5% aqueous KOH; individual hyphae (29)33–60(125) × (4)5–12(13) μm, cylindrical, thin-walled, branching frequently, secondary septation absent. ***Subhymenium*** up to 10 μm thick. ***Stipitipellis*** composed of a tightly packed mass of subparallel, narrow, cylindrical hyphae, yellowish-brown in 5% aqueous KOH; individual hyphae (33)37–79(102) × 4–13(16) μm, thin-walled, hyaline to pale yellow in 5% aqueous KOH, branched frequently, secondary septation absent. ***Clamp connections*** absent.

##### Habitat.

Scattered or gregarious in small clusters on the ground in coniferous and angiosperm mixed forests.

#### ﻿ Craterellus striatus

Taxon classificationFungiCantharellalesCantharellaceae

G.P. Zhao, J.J. Hu, B. Zhang & Y. Li sp. nov.

469F80AF-45B6-56DD-8C5B-95D80C31644C

 842531

Figs 4 , 5

##### Holotype.

China. Jilin Province, Baishan City, Fusong County, Quanyang Town, alt. 780.4 m, 42.23°N, 121.30°E, 22 August 2021, G. P. Zhao, J. J. Hu, and Bo Zhang (HMJAU 61463, GenBank Acc. nos.: nr LSU = OM509446, OM509447, *tef*-1α = ON125913, ON125914).

##### Etymology.

*Striatus*: referring to the fringe of pileus.

##### Diagnosis.

Differs from other *Craterellus* species by the fibrillose, greyish-brown to yellowish-brown, fully perforated pileus with a brown fringe, the hymenophore with a forking vein, not anastomosing and non-discolouring upon drying.

**Figure 4.**
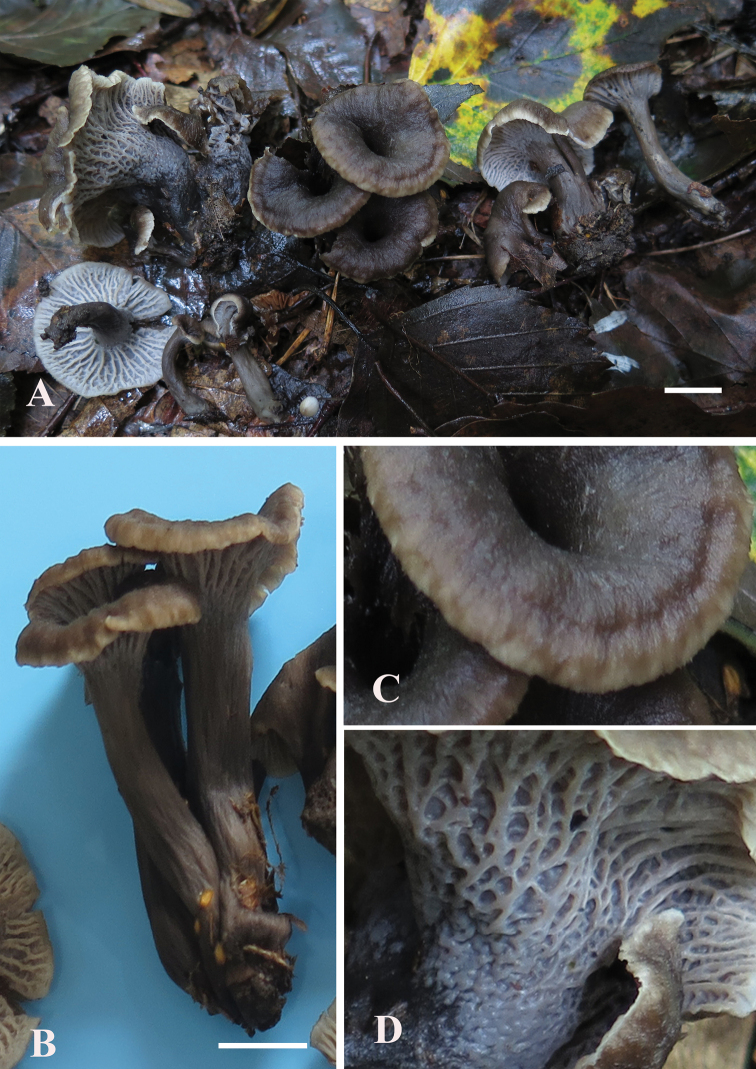
*Craterellusstriatus* (HMJAU 60412, holotype) **A** Fresh basidiocarps **B** Stipe **C** Pileus **D** Hymenophore. Scale bars: 1 cm (**A, B**)

##### Description.

***Fruiting body*** 12–45 mm high. ***Pileus*** 3–21 mm in diam., 1–10 mm tall, plane-convex firstly, soon infundibuliform and usually perforated continuous to the base of the stipe; margin inrolled when young, then expanding outwards, old becoming incurved, broadly wavy; surface blackish-brown, cream near the margin at first, gradually lighter to the centre with age, yellowish-brown finally, turning pale greyish-brown when drying, covered with brown fringe and fibrillose scales. ***Hymenophore*** decurrent, consisting of longitudinal ridges (<1 mm) with prominent forking, cross vein and off-white. ***Stipe*** 11–35 × 1–6 mm, cylindrical, inflated at the base, up to 10 mm in diam., hollow, central, smooth, blackish-brown. ***Text*** leathery, dirty white. ***Odour*** light. ***Taste*** unknown. ***Spore print*** not obtained.

**Figure 5.**
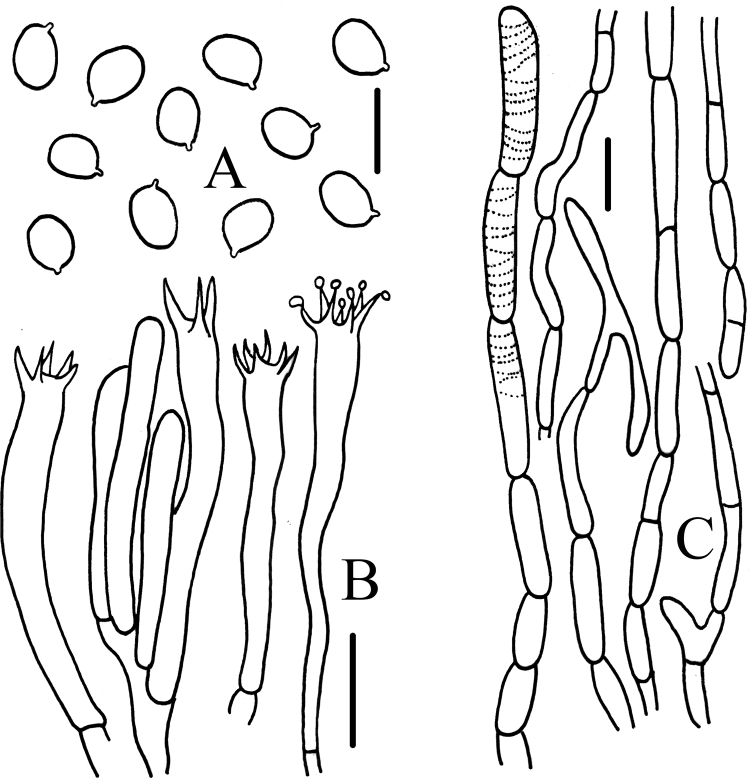
Microscopic characteristics of *Craterellusstriatus* (HMJAU 60412) **A** spores **B** basidiobasidia **C** pileipellis. Scale bars: 10 μm (**A**); 20 μm (**B**); 40 μm (**C**).

***Basidiospores*** (6.5)6.8–7.4–8.0(8.8) × (5.0)5.1–5.5–6.0 μm, Q = (1.18)1.22–1.36–1.53(1.73), Q_m_ = 1.36 ± 0.12, broad ellipsoid to ellipsoid, smooth, thin-walled, hyaline to pale yellow in 5% aqueous KOH, inamyloid. ***Basidia*** (34)40–67(68) × 6–9 μm, slender, sterigmata 2–6. ***Cystidia*** absent. ***Hymenium*** in transverse section 45 μm thick, yellowish-brown in 5% aqueous KOH. ***Pileipellis*** scarcely differentiated from trama, a cutis of largely cylindrical hyphae, yellowish-brown in 5% aqueous KOH; individual hyphae (30)40–70(73) × (4)5–14(15) μm, thin-walled, encrusted, branched frequently, secondary septation absent, hyaline in 5% aqueous KOH. ***Pileus trama*** up to 500 μm thick, subparallel, yellowish-brown in 5% aqueous KOH; individual hyphae (29)33–60(125) × (4)5–12(13) μm, cylindrical, thin-walled, encrusted, branching frequently, secondary septation absent. ***Subhymenium*** up to 10 μm thick. ***Stipitipellis*** composed of a tightly-packed mass of subparallel, narrow, cylindrical hyphae, yellowish-brown in 5% aqueous KOH; individual hyphae (33)37–79(102) × 4–13(16) μm, thin-walled, hyaline to pale yellow in 5% aqueous KOH, branched frequently, secondary septation absent. ***Clamp connections*** absent.

##### Habitat.

Scattered to gregarious on the ground in a coniferous and angiosperm mixed forest.

### ﻿Key to reported species of *Craterellus* in China

**Table:** 

1	Clamp connections present	**2**
–	Clamp connections absent	**4**
2	Stipe golden yellow to orangish-yellow	**3**
–	Stipe grey	***C.atratus***
3	Pileus perforate to the base of the stipe	***C.tubaeformis***
–	Pileus not perforate	***C.lutescens***
4	Basidiomata not brown, absolutely light colour	**5**
–	Basidiomata greyish-brown to blackish-brown	**7**
5	Pileus white	***C.albidus***
–	Pileus yellow	**6**
6	Pileus very small, usually < 10 mm in diam	***C.aureus***
–	Pileus large, usually > 90 mm in diam	***C.luteus***
7	Basidiospores 10–15 μm long, average length > 11 µm	**10**
–	Basidiospores 6–12 μm long, average length < 11 µm	**8**
8	Hymenophore smooth to slightly folded	**9**
–	Hymenophore with well-developed veins or gill-folds	***C.striatus***
9	Basidia long, up to 106 µm long	***C.badiogriseus***
–	Basidia short	**13**
10	Basidia with 2 sterigmata	**11**
–	Basidia with 2–4 sterigmata	**12**
11	Basidiospores broad, up to 11.5 μm wide	***C.macrosporus***
–	Basidiospores 2–4 μm wide	***C.cornucopioides***
12	Pileus surface smooth	***C.croceialbus***
–	Pileus surface often with darker brown raised scales	***C.squamatus***
13	Margin dirty white	***C.connatus***
–	Margin not dirty white	***C.atrobrunneolus***

## ﻿Discussion

In our study, two new species formed a sister relationship with *Craterellusatrobrunneolus*. These three species show close morphological and phylogenetic similarities with each other. All species share the brown pileus, grey hymenophore, hollow stipe, narrow basidia and absence of clamps. *Craterellusatrobrunneolus* was initially described in south-western China. It is characterised by a dark brown to brownish-grey colouration, convex to plano-convex pileus with shallow depression, but not perforated centre, smooth to slightly folded hymenophore, absence of clamp connections in all tissues, narrow basidia with 2–6 sterigmata and broad ellipsoid to subglobose basidiospores (Cao et al. 2021b). *Craterellusconnatus* is recognised in the field by the medium-sized, nearly fleshy basidiomata with the greyish-brown, fully perforated pileus, an off-white margin, strongly anastomosed, veined hymenophore and hollow stipe. Microscopically, it possesses broad ellipsoid to ellipsoid basidiospores (Q_m_ = 1.32 ± 0.09), slender basidia with (2)4–6 sterigmata and an absence of clamp connections. *Craterellusstriatus* is characterised by its small-sized basidiomata, blackish-brown pileus that turns yellowish-brown upon drying and is covered with brown fringe and spinous scales and off-white hymenophore consisting of longitudinal ridges (<1 mm) with prominent forking. It has a hollow and brown stipe and broad ellipsoid to ellipsoid basidiospores, 2–6 spored basidia, encrusted hyphae and the absence of the clamp connection.

*Craterellusatrobrunneolus* differs by its dark brown to almost black throughout, convex but not perforated pileus, while *C.connatus* possesses greyish-brown pileus with an off-white margin and *C.striatus* possesses yellowish-brown, perforated pileus with brown fringe. Microscopically, *C.atrobrunneolus* possesses broad ellipsoid to subglobose basidiospores, while *C.connatus* and *C.striatus* possess broad ellipsoid to ellipsoid basidiospores. In addition, *C.atrobrunneolus* have 2–6 spored basidia, while *C.connatus* have mostly 4–6 spored basidia. *Craterellusstriatus* have encrusted hyphae, while *C.atrobrunneolus* did not. Although the two new species have similar microscopic characteristics (spores and basidia), they are separated by their colouration of dried pileus and hymenophore and the configuration of hymenophore. *Craterellusstriatus* is smaller (both pileus and stipe) than *C.connatus*. Further, *C.striatus* has a hymenophore composed of the forked longitudinal ridge and non-discolouring upon drying, whereas *C.connatus* has an anastomosing veined hymenophore, which turns khaki upon drying. Morphology and phylogenetic analyses indicated that the two new species in this study are not conspecific.

However, *Craterellusatrobrunneolus* was not included in any subgenus in Cao et al. (2021a). According to our study, *C.atrobrunneolus*, *C.conatus* and *C.striatus* have formed an isolated branch. Further research is needed to obtain a more precise infrageneric classification. Additionally, this study suggests that China, especially north-eastern China, has considerable fungal diversity and possibly many endemic species.

## Supplementary Material

XML Treatment for Craterellus connatus

XML Treatment for Craterellus striatus
