# ﻿Corrigenda for: “Two new species of *Craterellus* (Cantharellales, Hydnaceae) with veined hymenophore from north-eastern China” published in MycoKeys 91: 97–111, doi: 10.3897/mycokeys.91.84730

**DOI:** 10.3897/mycokeys.92.91389

**Published:** 2022-09-13

**Authors:** Gui-Ping Zhao, Jia-Jun Hu, Yong-Lan Tuo, Yu Li, Bo Zhang

**Affiliations:** 1 Engineering Research Center of Edible and Medicinal Fungi, Ministry of Education, Jilin Agricultural University, Changchun, Jilin 130118, China Jilin Agricultural University Changchun China

It was because of our mistakes, we mixed the figures used in our manuscript, and after it was published, we attention that our figures were in the wrong place. Thus, we provide below the new figures with corrected information.

**Figure 2.**
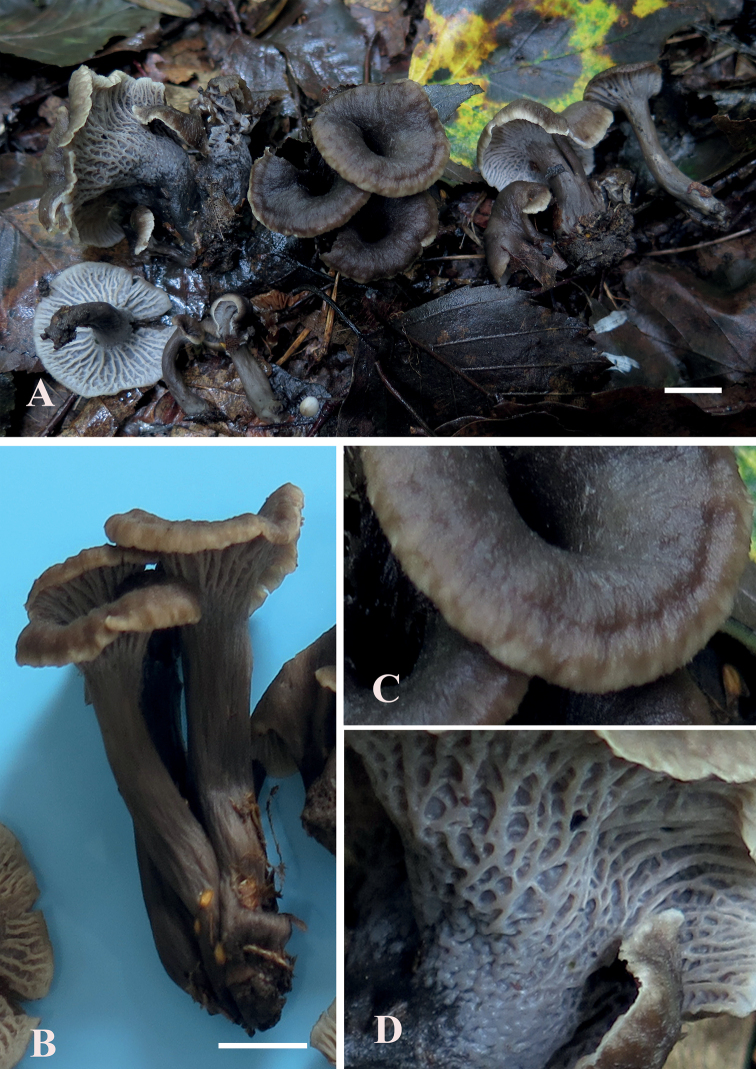
*Craterellusconnatus* (HMJAU 60411, holotype) **A** fresh basidiocarps **B** connate stipes **C** margin of pileus **D** hymenophore. Scale bars: 1 cm (**A, B**).

**Figure 4.**
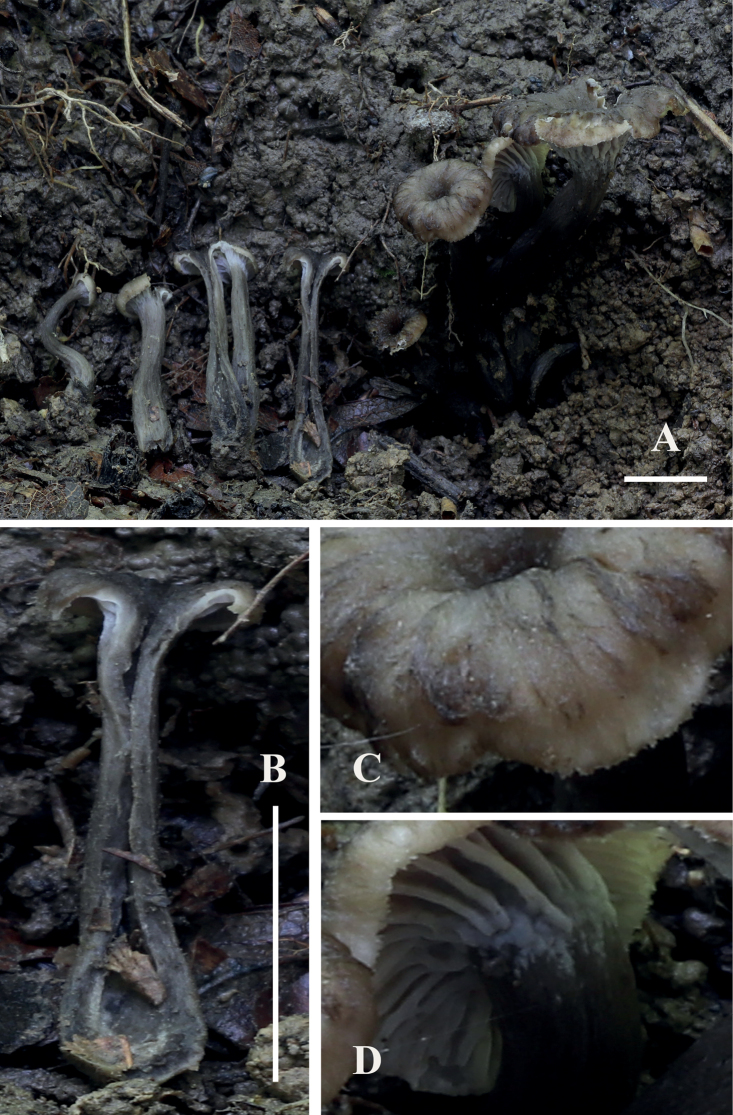
*Craterellusstriatus* (HMJAU 60412, holotype) **A** fresh basidiocarps **B** stipe **C** pileus **D** hymenophore. Scale bars: 1 cm (**A, B**).

